# Ketoconazole Shampoo for Seborrheic Dermatitis of the Scalp: A Narrative Review

**DOI:** 10.7759/cureus.67532

**Published:** 2024-08-22

**Authors:** Brynne E Tynes, Coplen D Johnson, Mayuri H Vaish, Brennan Abbott, Jelena Vučenović, Giustino Varrassi, Pooja Potharaju, Yair Lopez Torres, Zachary Lee, Shahab Ahmadzadeh, Sahar Shekoohi, Alan D Kaye

**Affiliations:** 1 School of Medicine, Louisiana State University Health Sciences Center, Shreveport, USA; 2 Department of Radiology, Louisiana State University Health Sciences Center, Shreveport, USA; 3 School of Medicine, University of Texas Southwestern Medical Center, Dallas, USA; 4 School of Medicine, American University of the Caribbean School of Medicine, Preston, GBR; 5 Department of Pain Medicine, Paolo Procacci Foundation, Rome, ITA; 6 Department of Anesthesiology, Louisiana State University Health Sciences Center, Shreveport, USA

**Keywords:** narrative review, topical antifungal, seborrheic dermatitis, ketoconazole shampoo, ketoconazole

## Abstract

Seborrheic dermatitis (SD) is a common inflammatory skin condition characterized by itchy, sensitive patches of greasy, flaky skin in areas rich in sebaceous glands, such as the scalp. Cases range from asymptomatic to debilitating, with effective treatment in severe cases proving crucial to patient quality of life. Ketoconazole shampoo is a topical antifungal that is a promising treatment option for individuals affected by this condition.Numerous trials display significant improvement in irritation and scaling of scalp seborrheic dermatitis (SSD) with ketoconazole shampoo treatment. Most studies also report optimally low relapse rates as well as little to no side effects, including rare skin irritation that resolves with cessation of the drug. Based on these findings, ketoconazole shampoo seems to be a safe and effective treatment for SSD.The present investigation reviews knowledge and research regarding ketoconazole shampoo as a treatment for SSD for physician consideration in the clinical setting.

## Introduction and background

Seborrheic dermatitis (SD) is an inflammatory skin condition characterized by patches of greasy flakes and scales in highly sebaceous areas, such as the scalp [1,2]. It is commonly seen in infancy and early adulthood and can present with itching or burning sensations and sensitivity in the affected region. The pathogenesis of SD is multifactorial, with the fungal species *Malassezia* being a significant contributing factor. It is usually part of the normal skin flora, but in some cases, it can irritate the production of lipases that hydrolyze lipids and trigger inflammatory processes on the skin [3]. SD may cause significant disruption and distress to the individual, so effective treatment is imperative [1]. Ketoconazole is an antifungal that inhibits fungal biosynthesis of phospholipids and triglycerides and alters sebum production, decreasing fungal growth and subsequent inflammation in SD [4,5]. The shampoo preparation of the drug is approved by the Food and Drug Administration (FDA) for dandruff, a milder skin condition, but it is still under investigation and has not yet been FDA-approved as a treatment for SD [6]. However, many studies have exhibited ketoconazole shampoo’s significant efficacy in improving scalp seborrheic dermatitis (SSD) symptoms with minimal side effects. The present investigation, therefore, aims to provide a comprehensive evaluation of the use of ketoconazole shampoo in SSD for physicians considering it as a treatment option for their patients. In this narrative review, we evaluate the background, symptoms, and diagnosis of SD, indications, pharmacology, results of existing trials, and side effects of ketoconazole shampoo for this condition.

## Review

Etiology, symptoms, diagnosis, and current treatment options for SD

Epidemiology

SD is a common inflammatory skin disease that manifests in areas rich in sebaceous glands. It has a papulosquamous morphology consisting of small, raised bumps and scales. A bimodal distribution occurs in infants, appearing during the first three months of life until around 12 months, then returning during puberty and early adulthood [1]. Additionally, SD is frequently diagnosed in older adults, particularly those with Parkinson’s disease (PD) [7].

The prevalence of SD is higher in males, likely related to higher sebum production than in females [8]. An epidemiological analysis in the United States found a prevalence of 4.11% among adults, which is comparable to results from other studies [9]. SD is also more prevalent in Caucasians compared to African Americans and Asian individuals. However, this disparity may be attributed to the under-recognition of clinical manifestations in individuals with darker skin tones [9].

Etiology

Adult seborrheic dermatitis (ASD) typically follows a relapsing and remitting course and is known to impair quality of life significantly [1]. Although the exact pathogenesis remains unclear, it is believed to be multifactorial, with factors including hormone levels, overactivity of sebaceous glands, fungal infection, environmental conditions, immunosuppression, and neuropsychiatric conditions. The most widely accepted theory points to the fungal species *Malassezia*, which thrives in sebum-rich areas. *Malassezia *is a lipophilic fungus that is part of the skin’s normal microbiota but can cause inflammation due to its ability to produce lipases that hydrolyze sebaceous lipids. This triggers keratinocytes to generate pro-inflammatory mediators through the arachidonic acid pathway, resulting in the clinical manifestations of ASD [3].

Environmental factors, such as low temperature, low UV index, and high humidity, can predispose individuals to ASD. Furthermore, it is more common in winter due to *Malassezia *spp.’s susceptibility to UV radiation and is similar to some fungal infections [10]. Immunosuppressed cases, such as those with human immunodeficiency virus (HIV), have an enhanced risk of developing SD. This is likely due to reduced levels of CD4 T-cells, leading to immune dysregulation and unchecked proliferation of *Malassezia* on the skin [11]. PD is another common risk factor for SD. Many genes associated with PD affect lipid metabolism, increasing intracellular lipid concentrations. Additionally, α-synuclein (SNCA) alleles commonly affected in PD make lipid droplets more permeable to lipases. These genetic factors allow *Malassezia *spp.’s over-colonization in patients with PD [12].

Signs and Symptoms

SD commonly presents with well-delineated patches of greasy, yellow, flaky scales in highly sebaceous areas, such as the scalp, nasolabial folds, and retro auricular region. While it is often asymptomatic, it can be associated with intense pruritis, burning sensations, and skin sensitivity. In individuals with lighter skin, these areas may appear erythematous. However, darker skin's affected areas may be hypopigmented or hyperpigmented without erythema [2]. Dandruff, a mild form of SD, typically presents subclinically with minor areas of erythema on the scalp. In African Americans, severe dandruff can manifest as hypopigmentation, which differs from its presentation in lighter-skinned individuals. Despite these differences, pruritis is a common symptom of dandruff across all skin types [2].

Diagnostic Criteria

SD is primarily diagnosed based on the individual’s medical history and clinical examination. Although there is no standardized diagnostic system for SSD, a recent study introduced a point scale to assess its severity. This scale considers factors such as adherent scalp flaking, maximal erythema area, and pruritus [13]. Additional diagnostic criteria like blood tests may be used when SD is extensive or severe and can include hepatitis C virus (HCV), zinc, riboflavin, pyridoxine, niacin, and essential fatty acid levels [14]. This approach is particularly relevant for cases with acquired immunodeficiency syndrome (AIDS) or other stages of HIV, as these populations are commonly affected. Dermatological onset in such individuals typically occurs when CD4 counts drop to 450-550 cells/microliter [14]. Dermatoscopy, a noninvasive technique that allows for magnified observation of the skin in vivo, is also used to diagnose SD. Common dermatoscopic features of SD include dotted vessels in a patchy distribution with fine white-yellowish scales, follicular plugs, and an orangish color. This technique helps differentiate SD from other conditions like psoriasis or tinea capitis [15].

Histological examination can further aid in diagnosing SD, particularly distinguishing between acute and chronic forms. In acute SD, histological features include spongiosis and psoriasiform hyperplasia in the epidermis, along with “shoulder parakeratosis” around follicular openings [16]. The dermis may show lymphocytes and histiocytes. Chronic SD is characterized by marked parakeratosis and psoriasiform epidermal hyperplasia, with possible dilation of superficial dermal venules [16]. Overall, the diagnosis of SD commonly involves only a clinical evaluation; however, laboratory tests, dermatoscopic examination, and histological examination may be used if necessary.

Current available treatment options

A variety of treatment options are available for SD. Lifestyle modifications, particularly increased sun exposure, can be beneficial. One prospective study demonstrated that UVB phototherapy is an effective and safe treatment for severe SD [17]. Several topical agents are available for treating SSD in adults, including ketoconazole, ciclopirox, miconazole, betamethasone valerate, zinc pyrithione, selenium sulfide, and clobetasol propionate [1]. Ketoconazole, ciclopirox, and miconazole possess antifungal properties that effectively reduce mean erythema, scaling, and itch in this condition, and recent studies have shown ketoconazole shampoo to be clinically effective in treating *Malassezia*-related conditions such as SSD [6,18]. While ketoconazole shampoo is generally safe, clinicians should know the potential for allergic contact dermatitis [6]. Topical corticosteroids, such as betamethasone valerate and clobetasol propionate, may be used for severe flares. These drugs provide quick relief from erythema, scaling, and pruritus through anti-inflammatory, immunosuppressive, and antiproliferative mechanisms [19-21]. Systemic drugs, such as oral terbinafine and itraconazole, are used for severe cases to begin maintenance with topical antifungals. Terbinafine has antimycotic properties against an array of fungi, including *Malassezia* spp. [22]. Itraconazole is a highly lipophilic antifungal secreted with sebum at sites where *Malassezia *spp. commonly reside [23]. Both systemic antifungals have been proven effective in clinical trials [24,25].

Ketoconazole pharmacology and clinical utility

Ketoconazole is a member of the imidazole family of antifungals that operates by inhibiting the production of lanosterol, a precursor for the biosynthesis of ergosterol, which normally maintains fungal membrane integrity [26]. This increases membrane fluidity and halts fungal growth. Moreover, it strongly binds to the cytochrome p450 mono-oxygenase complex, further hindering the fungal biosynthesis of triglycerides and phospholipids, including ergosterol [4]. In addition to its antifungal effects, ketoconazole has been shown to shift sebum secretion in the stratum corneum of patients with SD [5]. A recent study has suggested that it could have benefits beyond its antifungal activity by influencing the skin's lipid profile, decreasing sebum production, and altering *Malassezia *lipid metabolism [5]. Since toxicity to the stratum corneum from fungi such as *Malassezia furfur* can be attributed to their production of fatty acids and indoles, decreased availability of these lipids with ketoconazole treatment can improve SD symptoms. Additionally, the reduction of lipids favors the colonization of biotin-producing bacteria, such as *Cutibacterium acnes*, on the skin, which lowers inflammation and cell proliferation in SD patients. Ketoconazole is best absorbed systemically via the oral route. Oral absorption is variable due to its interaction with digestive contents, being reduced by antacids and increased by food or dilute hydrochloric acid [27]. In the blood, 83.7% of the drug is bound to plasma protein, primarily albumin, 15.3% to erythrocytes, and 1% unbound. Interestingly, ketoconazole shows strong distribution to the skin; a 200 mg oral dose produces a 0.91 mg/L concentration in suction blister fluid [28]. It also distributes well into the eyes, saliva, urinary tract, and tendon but does not cross the blood-brain barrier [29-32]. This explains its potent effects in dermatological conditions such as SD.

Oral ketoconazole likely has saturable hepatic first-pass metabolism and a two-compartment pharmacokinetic model of distribution [27]. It shows a dose-dependent half-life that increases with longer-term treatment, indicating autoinhibition of metabolism. It is primarily excreted in the feces after metabolization into inactive products. Topical preparations of ketoconazole show minimal systemic absorption and, therefore, do not elicit systemic effects. The shampoo form is normally prescribed at 2% concentration by weight. It shows higher drug concentration and bioactivity in the stratum corneum upon application than its counterpart antifungals, such as miconazole [33]. Furthermore, it exhibits constant, zero-order pharmacokinetics in the skin, as concentrations decrease linearly with time upon cessation of the drug. It also has a half-life of approximately 36 hours in the stratum corneum [33]. These properties make its effects long-lasting and ideal for cutaneous application through shampoos.

Given its antifungal and anti-inflammatory properties, ketoconazole is indicated primarily for pityriasis versicolor, dermatophytosis, and other conditions associated with tinea infections, *Malassezia* spp., and *Candida *spp. and is still being investigated as a potential treatment for androgenetic alopecia, acne, and SD [6]. Interestingly, despite differences in absorption, ketoconazole’s topical efficacy is similar to its oral administration [34,35]. The European Medicines Agency and the UK Government have banned oral ketoconazole use due to its side effects, including hepatotoxicity [18]. Therefore, topical preparations of ketoconazole provide adequate therapeutic value without the risks of side effects from systemic administration.

Ketoconazole shampoo for SD of the scalp

Indications for Ketoconazole Shampoo in SD

Ketoconazole shampoo is primarily indicated for active SD due to its antifungal properties, targeting and reducing the proliferation of *Malassezia *spp. Two concentrations of ketoconazole shampoo are also FDA-approved for the treatment of dandruff [36,6]. A 1% concentration is available over the counter, and a 2% concentration is available by prescription only. Additionally, prophylactically, it is indicated to prevent the recurrence of SD and dandruff in patients previously affected by these conditions [37].

Measuring Response to Ketoconazole Shampoo

Responses to ketoconazole shampoo have been assessed using various methods, one of the most common being clinical grading scales. These assessments vary by study, but common measures include erythema, dandruff, and scaling severity evaluated by clinicians using a numeric scale [37-39]. Squamometry has also been used, featuring adhesive discs applied to the scalp to collect skin flakes and cells [38,40]. These can be weighed and analyzed to objectively measure a treatment’s effects [38,40,41]. The relapse rate is also important; studies often have a follow-up period ranging from four weeks to many months after the treatment period has ended to assess long-term efficacy [37,38,40]. These methods comprehensively evaluate ketoconazole shampoo’s effectiveness in treating SD.

Clinical Efficacy

Many studies have demonstrated the efficacy of ketoconazole shampoo in improving the symptoms of SD. In one double-masked placebo-controlled study, 38 patients with SSD were treated with either ketoconazole or a placebo twice weekly for four weeks [39]. After the treatment period, 89% (16 of 18) of patients treated with ketoconazole experienced improvement or were free of lesions, compared to only 44% (8 of 18) of the placebo group (p < 0.01) [39]. In a similar double-masked study, 20 subjects with SD were treated with 2% ketoconazole or placebo shampoo two to three times weekly for four weeks [42]. Scalp scaling was significantly reduced in the ketoconazole group compared to the placebo group after two, three, and four weeks of treatment (p < 0.05) [42]. One comparative study differentiated the effectiveness of two shampoo concentrations in treating SD and severe dandruff by randomizing 66 patients into a 1% treatment or 2% treatment group [38]. Subjects applied the shampoo twice weekly for four weeks. While both shampoos were effective in reducing scalp flakiness and *Malassezia *spp. density, the 2% shampoo was significantly more effective at two weeks (p < 0.05) and four weeks (p < 0.01) of treatment. The 2% shampoo also showed fewer relapses (23%) than the 1% shampoo (39%), but this finding did not reach significance. These results suggest that higher concentrations of ketoconazole may be more beneficial in treating these conditions [38].

Ketoconazole has also been evaluated against various alternative treatments. In one randomized parallel-group trial, 331 subjects with severe SD or dandruff were treated with four weeks of ketoconazole 2% shampoo or zinc pyrithione 1% shampoo, followed by a four-week follow-up phase [40]. Beneficial effects were found in both shampoos, but a significantly greater effect was seen in the ketoconazole shampoo group, with a 73% improvement in the dandruff severity score compared to 67% in the zinc pyrithione shampoo at four weeks of treatment (p < 0.02). The researchers also found significantly decreased rates of recurrence of SD in the ketoconazole group compared to the zinc pyrithione group (p < 0.03). Results showed a significantly better overall clearing of the skin condition at the end of the study in the ketoconazole group (p = 0.004), indicating ketoconazole shampoo is not only effective but more efficacious than zinc pyrithione shampoo in treating severe cases of SD [40]. Other studies have found ketoconazole shampoo to be roughly equal in effectiveness as a combination anti-inflammatory/antifungal shampoo (piroctone olamine, *Vitis vinifera*, lactic acid, lactoferrin, dipotassium glycyrrhizate, telmesteine) and 2% miconazole nitrate shampoo in treating SD [43,44] (Table 1) [37-40,42,45-49].

**Table 1:** Summary of clinical trials on ketoconazole shampoo in treating SD or dandruff SD: seborrheic dermatitis

Author (year)	Subjects	Protocol	Findings/results	Adverse effects	Conclusions
Peter et al. (1995) [37]	575	Phase I: 2% ketoconazole shampoo, twice weekly for two to four weeks. Phase II: placebo or 2% ketoconazole once weekly or biweekly.	Phase I: SD was resolved in 88% of patients. Phase II: 19% of patients treated with ketoconazole weekly experienced relapse vs. 47% of patients treated with placebo.	Phase I: 6.4%. Phase II: 3.9% itching and burning were experienced most often. A contact dermatitis was reported in two patients.	Ketoconazole 2% shampoo is effective in clearing and preventing relapses of SD.
Piérard-Franchimont (2001) [38]	66	2% ketoconazole shampoo vs. 1% ketoconazole shampoo, twice weekly for four weeks.	Both shampoos were effective in reducing scalp flakiness, and *Malassezia *spp. density. The 2% concentration was more significantly effective at two and four weeks of treatment (p < 0.05).	One patient in the 1% ketoconazole group experienced seborrhea. Other adverse effects were mild and not specifically mentioned.	2% ketoconazole shampoo has superior efficacy to 1% ketoconazole in treating SD and severe dandruff.
Faergemann (1990) [39]	36	Ketoconazole shampoo vs. placebo shampoo, twice weekly for four weeks.	89% of patients in the ketoconazole group improved or became free of lesions vs. 44% in the placebo group (p < 0.01).	No adverse effects were found.	Ketoconazole shampoo is safe and effective in treating SSD.
Piérard-Franchimont (2002) [40]	331	2% ketoconazole shampoo vs. 1% zinc pyrithione shampoo, twice weekly for four weeks.	The ketoconazole group had 73% improvement in dandruff severity vs. 67% in zinc pyrithione group (p < 0.02). The recurrence of SD or severe dandruff was also significantly lower in the ketoconazole group.	In the ketoconazole group, two patients had itching, and one patient had erythema. In the zinc pyrithione group, two patients had erythema.	2% ketoconazole shampoo was more effective than 1% zinc pyrithione shampoo in the treatment of SD or severe dandruff.
Green et al. (1987) [42,46]	20	2% ketoconazole shampoo vs. placebo shampoo, two to three times weekly for four weeks.	Scalp scaling was significantly reduced in the ketoconazole group compared to the placebo after two, three, and four weeks of treatment (p < 0.05).	No adverse effects were found.	2% ketoconazole shampoo is effective in treating SD.
Buechner et al. (2013) [44]	274	2% ketoconazole shampoo vs. 2% miconazole nitrate shampoo, twice weekly for four weeks.	The Symptom Scale of Seborrheic Dermatitis, which measured erythema, scaling, and itching, was not significantly different between groups.	11.6% of patients in the ketoconazole group experienced adverse events vs. 15.2% in the miconazole group. Most adverse events were mild or moderate.	Both treatments are equally effective and tolerable in treating SD.
Dobrev et al. (1997) [45]	20	Uncontrolled study utilizing 2% ketoconazole shampoo, twice weekly for four weeks.	Significant improvements were seen in scalp scaling severity (p < 0.001).	No adverse effects were found.	2% ketoconazole shampoo is effective against *Pityrosporum *(*Malassezia*) yeasts.
Ratnavel et al. (2007) [47]	350	2% ketoconazole shampoo vs. 1.5% ciclopirox olamine shampoo vs. placebo shampoo, three times weekly for four weeks.	The scalp area affected by SD decreased with all treatments; however, both treatment groups showed significantly larger improvements than the placebo group from day 15 onward. The ciclopirox group had greater improvement than the ketoconazole group.	Two subjects in the ketoconazole group had skin irritation, and one had eye stinging. Two subjects in the ciclopirox group had burning skin sensations.	Ciclopirox olamine shampoo is as effective as ketoconazole shampoo, and both are superior to placebo in treating SD.
Squire et al. (2002) [48]	154	2% ketoconazole shampoo (Nizoral) vs. 1.5% ciclopirox olamine and 3% salicylic acid combination shampoo, three times weekly for four weeks.	Both groups showed significantly improved clinical and self-assessment scores and reduced effects of dandruff and SD following their respective treatments. There were no significant differences between the two groups.	A total of 36 adverse events were reported in the combination shampoo group, and 19 adverse events were reported in the ketoconazole group. The most common adverse effects were pruritis, rhinitis, and seborrhea.	The combination shampoo was effective and comparable with 2% ketoconazole shampoo in the treatment of SD and dandruff.
Chaijan et al. (2018) [49]	90	2% ketoconazole shampoo and placebo solution vs. *Myrtus communis* L. solution and placebo shampoo, eight times over four weeks.	Both groups showed significant improvement in pruritis, erythema, scaling severity, and area of scalp involvement. There were no significant differences between the groups.	Adverse events were observed in eight patients. There was no mention of specific events.	The *Myrtus communis *L. solution is effective for dandruff treatment and may be a substitute for 2% ketoconazole shampoo.

Adverse Effects of Topical Ketoconazole

Topical ketoconazole may cause several dermatological reactions, including but not limited to pruritis, dryness, stinging, and tingling at the application site [50]. When used in shampoo, it may alter hair texture or cause dry or oily hair and scalp. However, any skin irritation usually resolves with cessation of the drug. Less common adverse effects include alopecia, hypersensitivity reactions, impetigo, angioedema, contact dermatitis, and headache. Therefore, it is contraindicated in any person with a history of hypersensitivity reactions or skin irritation with ketoconazole [50].

## Conclusions

SD is a frequently occurring inflammatory skin condition appearing as a papulosquamous rash in regions with abundant sebaceous glands, notably the scalp, face, and skin folds. It is characterized by patches of greasy flakes that may be asymptomatic or present with pruritis and irritation. It ranks third among dermatological conditions regarding its impact on a patient's quality of life, following atopic and contact dermatitis. Therefore, symptom control for this condition is extremely important. Management of SD mainly consists of topical treatments to minimize adverse effects. Ketoconazole shampoo has been proven to be safe and effective in treating and preventing the symptoms of SD. Although not FDA-approved for this indication, clinicians frequently prescribe it with great success. Physicians should avoid use in patients with previous reactions to ketoconazole and monitor for skin irritation in all individuals. In addition to an efficient treatment regimen, patients should be counseled to minimize exposure to triggers that worsen SD symptoms and avoid irritating behavior such as excessive scratching. In all cases, it is essential to implement a multidisciplinary approach personalized to each patient to ensure the best possible outcomes and quality of life.
